# Fatty acid uptake in *Trypanosoma brucei*: Host resources and possible mechanisms

**DOI:** 10.3389/fcimb.2022.949409

**Published:** 2022-11-21

**Authors:** Nava Raj Poudyal, Kimberly S. Paul

**Affiliations:** ^1^ Department of Genetics and Biochemistry, Clemson University, Clemson, SC, United States; ^2^ Eukaryotic Pathogens Innovation Center (EPIC), Clemson University, Clemson, SC, United States

**Keywords:** *Trypanosoma brucei* (*T. brucei*), fatty acids, uptake, lipids, host-parasite interactions, metabolism

## Abstract

*Trypanosoma brucei* spp. causes African Sleeping Sickness in humans and nagana, a wasting disease, in cattle. As *T. brucei* goes through its life cycle in its mammalian and insect vector hosts, it is exposed to distinct environments that differ in their nutrient resources. One such nutrient resource is fatty acids, which *T. brucei* uses to build complex lipids or as a potential carbon source for oxidative metabolism. Of note, fatty acids are the membrane anchoring moiety of the glycosylphosphatidylinositol (GPI)-anchors of the major surface proteins, Variant Surface Glycoprotein (VSG) and the Procyclins, which are implicated in parasite survival in the host. While *T. brucei* can synthesize fatty acids *de novo*, it also readily acquires fatty acids from its surroundings. The relative contribution of parasite-derived vs. host-derived fatty acids to *T. brucei* growth and survival is not known, nor have the molecular mechanisms of fatty acid uptake been defined. To facilitate experimental inquiry into these important aspects of *T. brucei* biology, we addressed two questions in this review: (1) What is known about the availability of fatty acids in different host tissues where *T. brucei* can live? (2) What is known about the molecular mechanisms mediating fatty acid uptake in *T. brucei*? Finally, based on existing biochemical and genomic data, we suggest a model for *T. brucei* fatty acid uptake that proposes two major routes of fatty acid uptake: diffusion across membranes followed by intracellular trapping, and endocytosis of host lipoproteins.

## 1 *Trypanosoma brucei* life cycle and coat proteins

*Trypanosoma brucei* spp. are protozoan parasites transmitted by tsetse flies (*Glossina* ssp.) that cause lethal disease in humans, called African Sleeping Sickness or Human African Trypanosomiasis (HAT), and a wasting disease in wild animals and livestock, called nagana or Animal African Trypanosomiasis (AAT) (Morrison et al., 2016; Odeniran et al., 2018). The *T. brucei* life cycle alternates between the mammalian host (animal or human) and the tsetse fly (Wamwiri and Changasi, 2016). In the mammalian host, *T. brucei* dwells extracellularly in the blood and lymph (bloodstream form, BSF), where they derive energy from glucose metabolism. *T. brucei* also can cross the blood-cerebrospinal fluid (CSF) and blood-brain barriers to invade the central nervous system (Wolburg et al., 2012; Mogk et al., 2017), as well as harbor in the interstitium of tissues such as adipose, skin, and testes (Capewell et al., 2016; Trindade et al., 2016). In a mouse model of infection, *T. brucei* adipose-dwelling BSFs displayed alterations to their transcriptome, including upregulation of lipid metabolism and fatty acid β-oxidation genes, that suggested a metabolic adaptation to the adipose environment (Trindade et al., 2016). A quorum-sensing pathway triggers proliferating BSFs to transform to non-proliferating short stumpy forms (SSFs), which are hypothesized to be an adaptive mechanism to control parasite load (Mony et al., 2014; Rojas and Matthews, 2019; Cayla et al., 2020). Historically, it was thought that only SSFs were competent to infect the tsetse fly, primarily due to pre-adaptive metabolic changes that occur upon SSF differentiation (Rico et al., 2013). However, more recently it was shown that BSFs are also capable of establishing infections in flies (Schuster et al., 2021). In terms of transmission to flies, *T. brucei* in skin, adipose, and blood, all have been shown to be competent sources for tsetse infection (Capewell et al., 2016; Trindade et al., 2016). Tsetse flies are obligate blood-feeders, acquiring and transmitting the parasites when they take a blood meal. Upon their ingestion by the fly, BSFs and SSF differentiate into proliferative midgut procyclic forms (PCFs) (Schuster et al., 2021), where they rely on catabolism of proline and other amino acids (Millerioux et al., 2013; Mantilla et al., 2017). In the fly midgut, PCFs cross the peritrophic matrix to enter the ectoperitrophic space and migrate to the proventriculus in the foregut, where they differentiate into epimastigote forms (Rose et al., 2020). The epimastigotes then migrate to the salivary glands and subsequently differentiate into infective trypomastigotes called metacyclics, which are the mammalian infective forms.

Because *T. brucei* is an extracellular parasite, it is fully exposed to the host immune system and other defense mechanisms. Thus, the surface protein coats of *T. brucei* play important roles in protecting the parasite while it is in its hosts. Procyclins are a small five-gene family of surface coat glycoproteins expressed by *T. brucei* in the tsetse fly (Mowatt and Clayton, 1987; Roditi et al., 1987; Richardson et al., 1988; Acosta-Serrano et al., 1999). Though not essential in culture, procyclins are required for efficient colonization of the tsetse fly (Ruepp et al., 1997; Nagamune et al., 2000; Güther et al., 2006). The C-terminal domains of procyclins appear to be protease-resistant and so may provide protection against proteolytic attack in the tsetse midgut (Acosta-Serrano et al., 2001). Variant Surface Glycoproteins (VSGs) are expressed in *T. brucei* in the mammalian host from a large repertoire of ~2500 genes (Cross et al., 2014). The tsetse fly salivary gland metacyclic forms also have VSG coats, which are expressed from a set of metacyclic-specific VSG genes (Hutchinson et al., 2016; Kolev et al., 2017). The highly immunogenic VSG coat, consisting of 10^7^ copies of one VSG variant, is essential for survival inside the mammalian host (Turner et al., 1985). The VSG coat mediates two major mechanisms to evade the host immune response. The first mechanism is antigenic variation, in which the parasite switches its VSG coat to a new variant that avoids detection by the existing pool of anti-VSG antibodies. Antigenic variation enables population-level escape from the adaptive immune response (Cross et al., 2014). The second mechanism is the rapid endocytic recycling of VSG. Surface VSGs bound to antibodies and complement are rapidly internalized into endosomes, where the serum proteins are routed to the lysosome for degradation and the unbound VSG is returned to the surface (Pal et al., 2003; Engstler et al., 2007). This rapid endocytic turnover was proposed to “scrub” host immune proteins from the parasite’s surface, enabling *T. brucei* to escape phagocytic clearance by the innate immune response (O’Beirne et al., 1998; Allen et al., 2003; Engstler et al., 2007). Supporting this idea, when VSG endocytic recycling was slowed by RNAi of the kinesin KIFC1, these RNAi parasites exhibited reduced infectivity *in vivo* (Lecordier et al., 2020). This process would be particularly important to parasites that had recently switched their VSG coats and thus, were still displaying the prior VSG on the surface.

The Procyclin and VSG coat proteins are attached to the plasma membrane *via* a covalently linked glycosylphosphatidylinositol (GPI)-anchor inserted into the outer leaflet of the lipid bilayer. GPI-anchors have a conserved core structure consisting of an ethanolamine phosphate attached to a Man_3_GlcN core glycan linked to phosphatidylinositol (Morita et al., 2000; Nagamune et al., 2000; Lillico et al., 2003; Smith and Bütikofer, 2010; Kinoshita and Fujita, 2016). GPI-anchors are synthesized in the endoplasmic reticulum (ER) and added to the protein C-terminus during translation, as in other eukaryotes (Ferguson et al., 2009; Yadav and Khan, 2018). The Procyclin and VSG GPI-anchors differ in the structure of their glycan side chains, and in the arrangement and composition of their fatty acids (Hong and Kinoshita, 2009). While the fatty acids found in the Procyclin GPI-anchor are abundant C16 and C18 species (Field et al., 1991), the fatty acids in the VSG GPI-anchor are exclusively C14:0 (myristic acid) (Ferguson and Cross, 1984; Ferguson et al., 1985). The GPI-anchors are critical to the coat proteins for their efficient secretory and endocytic trafficking in the secretory and endocytic pathways (McDowell et al., 1998; Grünfelder et al., 2002), for their proper targeting to the plasma membrane (Triggs and Bangs, 2003), and for their lateral mobility within the membranes (Buelow et al., 1988).

## 2 Fatty acids: A key nutrient for *T. brucei*

As parasites, *T. brucei* can acquire a variety of micro- and macronutrients directly from their hosts, including lipids such as fatty acids. Fatty acids have a variety of functional roles in addition to being major structural components of cellular membranes. In other organisms, fatty acids can serve as an energy source. *T. brucei* can carry out β-oxidation of fatty acids, but it is unclear if they are a significant source of energy (Dixon et al., 1971; Wiemer et al., 1996; Allmann et al., 2014; Trindade et al., 2016; Smith et al., 2017). Fatty acids also act as signaling molecules. Arachidonic acid (C20:4) stimulates intracellular Ca^+2^ release in *T. brucei* and other trypanosomes (Eintracht et al., 1998; Catisti et al., 2000), while C18:0 (stearic acid) stimulates the phosphorylation and inactivation of acetyl-CoA carboxylase (ACC), a fatty acid synthesis enzyme, in PCFs (Ray et al., 2018). Finally, fatty acids are key components of the coat protein GPI-anchors and, as the membrane-inserting moiety, are likely a major determinant of the dynamic properties of Procyclin and VSG coats in terms of their trafficking and lateral mobility in membranes.

As *T. brucei* goes through its life cycle, the availability of host fatty acids, both in kind and quantity, differs depending on the host and the tissue. Likewise, the fatty acid needs of *T. brucei* will depend on its developmental stage and what host niche it is occupying. For example, the fatty acid resources are likely high in adipose tissue, moderate in blood, but much lower in CSF. In terms of *T. brucei* itself, the dense VSG coat, consisting of 10^7^ VSGs/cell (Turner et al., 1985) each with a GPI-anchor bearing two myristate fatty acids (Ferguson et al., 1985), creates a high demand for myristate in BSFs. In contrast, the Procyclin coat has GPI-anchors requiring a mixture of longer C16 and C18 fatty acid species and is an order of magnitude less dense (3 x 10^6^ Procyclins/cell) (Acosta-Serrano et al., 1999).

## 3 Meeting fatty acid demands

*T. brucei* has two ways of meeting its fatty acid demands: it can make them itself or obtain them from the host. *T. brucei* lacks a typical cytosolic fatty acid synthase, so it synthesizes its own fatty acids *de novo via* two pathways: 1) an ER-localized fatty acid elongation pathway that produces fatty acids up to 22 carbons long and accounts for 90% of total fatty acid synthesis, and 2) a bacterial-type mitochondrial fatty acid synthesis pathway that produces primarily C8:0 (octanoic acid) and C16:0 (palmitic acid) and accounts for the remaining 10% of total fatty acid production in the cell (S. H. Lee et al., 2006; Livore et al., 2007; Stephens et al., 2007). Both fatty acid synthesis pathways are essential in *T. brucei* (Guler et al., 2008; S. H. Lee et al., 2006). For more information on the molecular mechanisms of fatty acid synthesis in *T. brucei*, please consult prior reviews (van Grinsven et al., 2009; Smith and Bütikofer, 2010; Uttaro, 2014; Parreira de Aquino et al., 2021). Despite its capacity for *de novo* fatty acid synthesis, *T. brucei* readily acquires fatty acids from its environment, likely because fatty acid synthesis is an energy-intensive process requiring two NADPHs and two ATPs per two carbons added. Thus, *T. brucei* can conserve significant energy by taking up fatty acids from its hosts rather than synthesizing them.

We propose that fatty acid synthesis is balanced with uptake in *T. brucei*. Where possible, *T. brucei* will use the “parasitic” option and acquire its fatty acids from the host. However, when the host supply is insufficient, then *T. brucei* synthesizes its own fatty acids to make up the difference and ensure its needs are met. One example of this comes in BSFs, which have a high demand for C14:0 myristic acid for the GPI-anchors of VSG and other membrane proteins. Myristic acid is relatively scarce in blood, so the BSF fatty acid synthesis pathway is specialized to make myristic acid, which it quantitatively incorporates into GPI-anchors (Morita et al., 2000). Meanwhile, the BSFs acquire other, more abundant, fatty acids by uptake from the environment (Paul et al., 2001; Lee et al., 2007). Indirect evidence for this balance between synthesis and uptake comes from the observation that BSFs in lipid-deprived conditions no longer synthesized primarily myristic acid, but instead synthesized longer fatty acids (Doering et al., 1993). Consequently, synthesis and uptake are two sides of the fatty acid acquisition coin for *T. brucei*. In the above example, it is the uptake of the abundant host fatty acids that enables the parasite’s synthesis machinery to focus on producing myristic acid, the one fatty acid the host is unable to adequately supply (Paul et al., 2001). Moreover, this balance must necessarily adjust as the parasite moves through its life cycle and through the different tissues of its tsetse fly and mammalian hosts.

## 4 Fatty acid resources in host tissues

**Figure 1:**
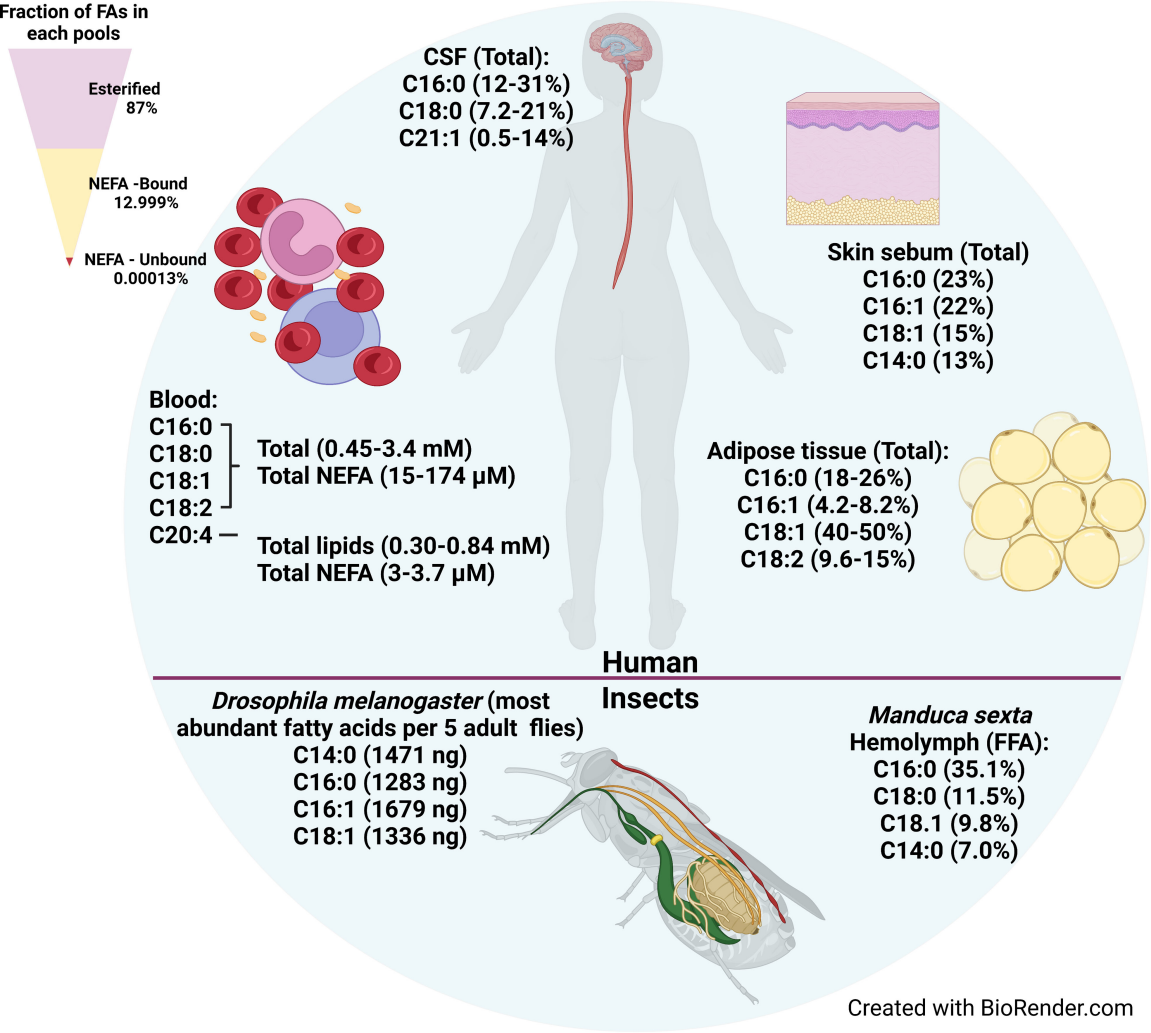
Fatty acid availability in human tissues, hemolymph, and insect bodies. The most abundant fatty acids available in various tissues where *T. brucei* harbors in human body are shown on upper portion. The most abundant fatty acids in the whole body of *Drosophila melanogaster* (fruit fly) and the most abundant fatty acids available in hemolymph of *Manduca sexta* (moth) are shown in the bottom portion. The fatty acids availability in tsetse fly was not found in the literature. Upper left triangle shows relative proportion in serum of esterified fatty acids, and the bound and unbound fractions of non-esterified fatty acids.

*T. brucei* can scavenge free fatty acids, lysophospholipids, lipids bound to proteins, and lipids in lipoprotein particles from their environment (Smith and Bütikofer, 2010; Parreira de Aquino et al., 2021). Here, we will review what is known about fatty acid resources in mammals and insects (**Figure 1**). Of note, there is no single standard for quantifying fatty acids in tissues, so inconsistencies in the units used for quantification makes it challenging to compare fatty acid concentrations across studies. We have tried to provide rough comparisons of fatty acid abundances and concentrations where possible. For example, we used the following values for the density of CSF (1.00059 g/mL) (Lui et al., 1998), the density of adipose (0.905 g/mL) (Martin et al., 1994), and the composition of adipose (80% fat/5% protein/15% water) (Abe et al., 2021) to convert the reported moles/weight and weight/weight values to µM values. **Figure 1** summarizes the most abundant fatty acids in each host tissue.

### 4.1 Fatty acid resources in mammals

#### 4.1.1 Fatty acid availability in serum

Serum is an abundant source of fatty acids. Serum fatty acids are present either in esterified form as components of more complex lipids, or in non-esterified form. In turn, the non-esterified fatty acid (NEFA) pool is divided between the unbound NEFA fraction, and the fraction bound to proteins, primarily albumin. Based on a LIPID MAPs Consortium study of human plasma, 87% of total plasma fatty acids are in the esterified form as components of glycerophospholipids, sterol esters, and glycerolipids (diacyl- and triacylglycerols), which account for 43%, 24%, and 20% of total serum lipids (% mg/dl), respectively (Quehenberger et al., 2010) (**Figure 1**, upper left triangle). The remaining 13% of plasma fatty acids constitute the total NEFA pool (Quehenberger et al., 2010). Part of the NEFA pool is associated with serum lipoproteins, where NEFAs make up a relatively small fraction of the lipoproteins’ total fatty acid content. However, the NEFA content of lipoproteins reported in the literature varies widely. One study showed that NEFAs were 1.1 - 3.4% and 1.7-3.2% of the Low Density Lipoprotein (LDL) and High Density Lipoprotein (HDL) fractions, respectively, which together comprised ~1.6% of total serum NEFAs (Skipski et al., 1967). A second study showed that 31-32% of the total serum NEFA pool is lipoprotein-associated, divided between Very Low Density Lipoproteins (VLDLs)/chylomicrons (CMs) (18.9-13.4%), LDLs (4.4-13.1%), and HDLs (5.9-8.1%) (Chung et al., 1995). The wide range of reported values may be due in part to technical artifacts during lipoprotein isolation that cause NEFAs to dissociate from albumin and partition into the lipoproteins (Goodman and Shiratori, 1964).

The majority (>99%) of the serum NEFA pool that is not associated with lipoproteins is bound to Albumin (Goodman, 1958; Spector et al., 1965; Richieri and Kleinfeld, 1995) (**Figure 1**, upper left triangle). Albumin is the most abundant serum protein, with a normal concentration of ~600 µM (ranging 500-1000 µM) and constituting 50-60% of total serum protein (Simard et al., 2006; van der Vusse, 2009; De Simone et al., 2021). Under normal physiological conditions the fatty acid:albumin molar ratio is 0.5–1, though albumin may carry up to nine fatty acids under some conditions (e.g. fasting) (Brodersen et al., 1990; Curry et al., 1998; van der Vusse, 2009). Albumin’s abundance and its high affinity for fatty acids (e.g. K_1_ of 0.5–2.5 µM for palmitate binding at the highest affinity site of human serum albumin) drives the buffering power of albumin such that it binds nearly all the free NEFAs in the serum and tissue fluids (van der Vusse, 2009). Thus, while the total NEFA pool ranges in concentration from 100 µM to >1mM, a study using the LIPID MAPs Consortium standard reference human plasma showed that the concentration of unbound, free NEFAs in plasma was very low, with the free unbound pool estimated to be < 10^-5^ of the total NEFA pool (on the order of 1-10 nM) (Huber and Kleinfeld, 2017) (**Figure 1**, upper left triangle). However, fatty acids also rapidly dissociate from albumin (with rates on the order of 2–6 sec^–1^) and subsequently transfer to acceptor sites, such as cellular membranes and fatty acid binding proteins at the cell surface (Massey et al., 1997; Demant et al., 2002). A variety of studies investigating fatty acid transport across membranes demonstrated that fatty acid dissociation from albumin is not rate-limiting for their transport across membranes (Kleinfeld et al., 1998; Demant et al., 2002; Carley and Kleinfeld, 2009; Vusse et al., 2022). Thus, free fatty acids bound to albumin constitute a highly bio-available and bioactive fraction of the NEFA pool.

Human serum concentrations of fatty acids differ depending on the person, their age, sex, diet, and any disease conditions (Bradbury et al., 2011; Wolters et al., 2014; Abdelmagid et al., 2015; Gallego et al., 2019). The most abundant fatty acids in total serum lipids and in the NEFA pool are C16:0 (palmitic acid), C18:0 (stearic acid) C18:1 (oleic acid), C18:2 (linoleic acid), and 20:4 (arachidonic acid) (Edelstein, 1986; Sera et al., 1994; Quehenberger et al., 2010; Gallego et al., 2019) (**Figure 1**, upper). Together these five most abundant fatty acids comprise ~90% of plasma NEFAs (Edelstein, 1986; Quehenberger et al., 2010). The same five fatty acids are also the most abundant in bovine sera, similarly accounting for ~ 90% of total serum lipids (Stoll and Spector, 1984). In terms of actual concentrations, studies found that in human total plasma lipids, the concentrations of the four most abundant saturated and unsaturated fatty acids (C16:0, C18:0, C18:1, and C18:2) were 0.45–3.4 mM (total serum lipids) and 15–174 µM (NEFA pool) (**Figure 1**, upper), while the concentrations of the polyunsaturated fatty acid 20:4 were lower, 0.30 - 0.84 mM (total lipids) and 3–3.7 µM (NEFA pool) (Sera et al., 1994; Quehenberger et al., 2010; Cunnane et al., 2012; Hellmuth et al., 2012; Abdelmagid et al., 2015; Christinat et al., 2016; Gallego et al., 2019).

Compared to the long chain C16 and C18 fatty acids, the medium chain saturated fatty acids such as C12:0 (lauric acid) and C14:0 (myristic acid) are much less abundant. C12:0 and C14:0 account for 0.4% and ~2–5.6%, respectively, of total plasma lipids by weight (Edelstein, 1986; Glaser et al., 2010; Bradbury et al., 2011). Similar values of 0.1-0.4% and 0.8–1.4% for C12:0 and C14:0, respectively, were found in bovine serum (Evans et al., 1961; Gregory et al., 2011). Plasma concentrations of C12:0 and C14:0 were less frequently reported. In the available studies, C12:0 concentrations were 5–50 µM in total lipids and 0.72–4.3 µM in the NEFA fraction, while C14:0 concentrations were higher, at 62–390 µM in total lipids and 6.1–17 µM in the NEFA fraction (Sera et al., 1994; Quehenberger et al., 2010; Cunnane et al., 2012; Hellmuth et al., 2012; Abdelmagid et al., 2015; Christinat et al., 2016; Gallego et al., 2019). Intra-study comparisons in the NEFA fraction of C12:0 and C14:0 with the four most abundant fatty acids (C16:0, C18:0, C18:1, and C18:2) indicated that C12:0 was 8.4—112X lower and C14:0 was 2.5—13.2X lower in concentration than the longer chain fatty acids (Quehenberger et al., 2010; Cunnane et al., 2012; Hellmuth et al., 2012; Christinat et al., 2016). Taken together, these studies show that in the bloodstream, *T. brucei* has access to substantial concentrations of long chain saturated and unsaturated fatty acids, many of which are present in total serum concentrations approaching the concentration of glucose (~ 5 mM), but has less access to medium chain fatty acids, whose concentrations tend to be lower. This may explain why *T. brucei* BSFs tend to synthesize primarily C14:0, the exclusive constituent of the BSF GPI-anchor, as there is much less availability in the host.

#### 4.1.2 Fatty acid availability in cerebrospinal fluid

Second stage HAT is marked by invasion of *T. brucei* into the central nervous system (CNS), as revealed by parasite detection in the CSF. CSF is considered a poor resource for fatty acids. The total fatty acid content found in the CSF is only ~0.54% of that found in the plasma (Koch et al., 2001). In one study, the concentrations of NEFAs, diacylglycerols (DAGs), and triacylglycerols (TAGs) was 9 µM, 0.20 µM, and 0.18 µM, respectively (Hanson et al., 2020). Like CSF fatty acids, the CSF albumin content relative to serum is similarly low (0.52% or 3-4 µM (Pisani et al., 2012), but sufficient to bind most of the free NEFAs in CSF.

The most abundant fatty acids, comprising ~90% of total fatty acids in CSF were C16:0 (12-31%), C18:1 (7.2-21%), and C22:1 (0.5-14%), a similar fatty acid profile to serum, except for the enrichment of C22:1 seen in CSF (Jumpertz et al., 2012; Fonteh et al., 2014; Nogueras et al., 2019) (**Figure 1**, upper). In CSF, C14:0 was also relatively scarce, comprising only 0.53-1.3% of total fatty acids (Fonteh et al., 2014; Nogueras et al., 2019). In the CSF NEFA fraction, C16:0 had the highest concentration, estimated from reported weight/volume values, ranging from 1.1-2.5 µM, followed by C18:0 (0.74 µM), C18:1 (0.15-0.45 µM), and C20:4 (0.0089-0.375 µM) (Pilitsis et al., 2001; Fonteh et al., 2014). In contrast to its abundance in total lipids, non-esterified C14:0 is present at a similar concentration (0.70 µM) to the C18 lipids in the NEFA fraction (Pilitsis et al., 2001). CSF alone is insufficient to support growth of *T. brucei* BSFs *in vitro* (Wolburg et al., 2012), likely due to an overall reduced availability of nutrients (Stoop et al., 2010). Interestingly, even in such a nutrient poor environment, the parasite manages to reach similar cell densities as to that observed in blood, albeit transiently (Mogk et al., 2014). Because CSF is an immune-privileged tissue, one advantage to CNS invasion is the ability to escape immune pressure in the bloodstream (Wolburg et al., 2012; Mogk et al., 2017). Moreover, studies have shown that in the later stage of *T. brucei* infection, infiltration of immune cells into the CSF was seen over time, suggesting the CSF may become a more hostile environment for the parasites (Pentreath et al., 1992; Wolburg et al., 2012). Consequently, maintaining immune evasion competence also would be important for *T. brucei* survival in the CSF. In summary, CSF is a relatively nutrient-poor niche for *T. brucei*, and the parasite appears to lack sufficient biosynthetic or nutrient mobilization capabilities to compensate enough to sustain colonization, which may be why occupancy of the CNS is limited to transient and cyclical episodes (Wolburg et al., 2012).

#### 4.1.3 Fatty acid availability in adipose tissue

The robust trafficking of fatty acids in and out of adipocytes means that adipose tissue is a lipid rich environment, making it favorable for *T. brucei* to harbor in (Thompson et al., 2010). Studies in mice revealed that adipose tissue indeed is a major reservoir for *T. brucei*, with a total parasite burden in the fat tissue exceeding that of the blood (Trindade et al., 2016). The most abundant fatty acids in adipose tissue are C16:0 (18–26%), C16:1 (4.0–8.2%), C18:1 (40–50%), and C18:2 (9.6–15%) (Halliwell et al., 1996; Bysted et al., 1999; Sjögren et al., 2012; Badoud et al., 2017) (**Figure 1**, upper). Compared to plasma and CSF, the relative abundance of C18:0 (3.0–5.4%) is reduced in adipose tissue, but the relative abundances of C12:0 (0.38–1.3%) and C14:0 (2.5–4.7%) are similarly low. Interestingly, the concentration of C14:0 was ~30% higher in subcutaneous adipose tissue from lean subjects compared to obese subjects (Badoud et al., 2017).

Given that the bulk of adipocytes are given over to lipid storage, the fatty acids stored in adipose tissue represent a large potential resource for *T. brucei*. However, the adipose-dwelling BSFs live extracellularly in the interstitial fluid of the adipose tissue, and so only have access to extracellular fatty acids that are released by the adipocytes into the interstitial fluid. The concentrations of fatty acids in adipose tissue interstitial fluids have not been thoroughly investigated. One *in vitro* study, which quantified the extracellular release of fatty acids from mouse adipose tissue explants upon epinephrine-stimulated lipolysis, found that the extracellular NEFA concentration rose from 1.5 mM up to 46 mM in 20 min (Cushman et al., 1973). A second *in vivo* study in humans quantified adipose venous fatty acid concentrations resulting from fasting-stimulated lipolysis and found that the concentrations of C14:0, C16:0, C18:0, C18:1, and C18:2 were 49 µM, 260 µM, 73 µM, 440 µM, and 150 µM, respectively (Halliwell et al., 1996). Together, these two studies suggest that the adipose tissue interstitial NEFA resources likely exceed that in the bloodstream, particularly during fasting and other lipolytic conditions. Of note, the adipose venous concentration of C14:0 was ~10X higher than in serum (Halliwell et al., 1996). In terms of buffering the released NEFAs, the interstitial albumin concentration in adipose is significantly lower than in serum (only 15% or ~ 100 µM) (Ellmerer et al., 2000). This lower albumin concentration suggests that during lipolysis, the buffering capacity of albumin may be exceeded, resulting in local elevation of free NEFA concentrations in adipose. Thus, the available information suggests that BSFs in adipose have an ample supply of fatty acids, and having upregulated β-oxidation and other lipid metabolic genes, they may be primed to take advantage of this lipid-rich environment (Trindade et al., 2016; Parreira de Aquino et al., 2021).

#### 4.1.4 Fatty acid availability in the skin

Delivered by the tsetse fly bite, *T. brucei* enters the mammalian host through the skin, where the parasites are deposited into the dermis. In addition to its role as a delivery site, the skin also serves as a long-term tissue reservoir for the parasite in the host body, even in the absence of blood parasitemia (Caljon et al., 2016; Capewell et al., 2016; Reuter et al., 2021). Much like in adipose tissue, *T. brucei* BSFs dwelling in skin likely also adapt their metabolism to establish themselves in the skin; however, the details of such adaptations are still unknown (Smith et al., 2017). The skin possesses sebaceous glands that produce sebum, which is rich in lipids. The most abundant fatty acids in sebum collected from nasal skin was found to be C16:0 (23%), C16:1 (22%), C18:1 (15%), and C14:0 (13%) (Ní Raghallaigh et al., 2012) (**Figure 1**, upper). C14:0 makes up >10% of the NEFA and TAG fatty acid fractions in sebum (Akaza et al., 2014). In humans, the concentration of some skin fatty acids varied depending on the location of the skin (Ludovici et al., 2018). The concentration of C16:1 and C18:1 was 5-22X higher in chest and head compared to the arm, while C24:0 was higher on the chest and arm than the head. As in other tissues, albumin is present in the skin interstitial fluid, at concentrations ranging from 30-60% of serum (160-300 µM) (Kramer et al., 1986; Haaverstad et al., 1997; Wiig et al., 2000), which is likely to provide adequate buffering of free NEFAs in skin interstitium. In summary, sebaceous gland secretion and the presence of subcutaneous adipose tissues likely make the skin a relatively fatty acid-rich environment for *T. brucei* to harbor in.

### 4.2 FA resources in insects

#### 4.2.1 Fatty acids in the gut

The gut and hemolymph of insects contain fatty acids, lipid carrier proteins, and lipoprotein particles capable of providing lipid nutrients for the parasites living in these tissues during their life cycle. However, there is a dearth of information about the fatty acid resources in tsetse flies, specifically, and in adult insects generally. As an indirect indication of lipid availability in the gut, one study of lipid digestion in the tsetse fly *Glossina morsitans morsitans*, the vector of *Trypanosoma congolense* (Masumu et al., 2010), showed that it excreted one third of the lipids in the blood meal it ingested (Langley et al., 1987). Comparison of the composition of excreted lipid with the ingested lipid showed that the percentage of phospholipids and TAGs decreased while the percentage of monoglycerides, cholesterol, and NEFAs increased, likely through lipolytic and absorptive activities. Cholesterol esters, which comprised 27% of the ingested lipids, were absent in the excreta, while hydrocarbons that were not observed in ingested lipids made up nearly 20% of the excreted lipids (Langley et al., 1987). This observation suggests that the tsetse fly gut is a relatively lipid-rich environment with non-esterified fatty acids liberated from serum lipids available for trypanosomes as they initiate their life cycle in the fly host.

#### 4.2.2 Fatty acids in insect tissues

Little information is available about the lipid composition and utilization by tsetse flies, but other Dipterans have been investigated. In the fruit fly *Drosophila melanogaster*, the four most abundant fatty acids in adults by weight (per 5 flies) were C14:0 (1471 ng), C16:0 (1283 ng), C16:1 (1679 ng), and C18:1 (1336 ng) (Hamida et al., 2021) (**Figure 1**, lower). In a variety of mosquito species, the most abundant fatty acids in adults were C16:0 (3.8–10 mg), C16:1 (2.2–43 mg)), and C18:1 (4.4–22 mg), expressed as mg/g wet weight (Sushchik et al., 2013). In mosquitoes, the sum of the medium chain fatty acids (C10:0, C12:0, and C14:0) ranged from 0.23 to 1.9 mg/g wet weight. In general, C16:1 appears to be more abundant in flies than in humans. For example, C16:1 accounted for 16–50% of the fatty acids in the TAG fraction in mosquitoes, but only 6% of the TAG fraction in human serum (Edelstein, 1986; Sushchik et al., 2013).

#### 4.2.3 Fatty acids in hemolymph

Much like mammalian blood, fly hemolymph contains a variety of lipids. The hemolymph of the moth *Manduca sexta* contains DAGs (41.5 mg/ml) and NEFAs (0.67 mg/ml), with C16:0 (42.6%) and C18:1 (35.6%) as the major DAG fatty acids and C16:0 (35.1%), C18:0 (11.5%), and C18:1 (9.8%) as the major NEFAs (Soulages and Wells, 1994) (**Figure 1**, lower). In terms of estimated concentrations, these levels would be on the order of 67 mM DAGs assuming they were all dioleoylglycerol, and 2.4 mM NEFAs if they were all C18:1. Nearly all hemolymph lipids are carried by lipophorin (Lp), which forms lipoprotein particles that range in density depending on their protein and lipid content, the latter of which accounts for 40-50% of Lp weight (Chino, 1982). The ratio of proteins to lipids in the tsetse fly *G.m. morsitans* Lp is ~1:1 (Ochanda et al., 1991). Insect Lp function is comparable to vertebrate LDL and HDL in transporting lipids between tissues (Ximenes et al., 2015; Kluck et al., 2018; Ben-Aicha et al., 2020). There is evidence that trypanosomes can access lipids bound by Lp in its insect host. *In vitro* experiments in which *T. rangeli* cells incubated with [^32^P]Lp or [^3^H]Lp showed transfer of radioactivity to the parasites, while no transfer was observed using the second most abundant hemolymph protein vitellogenin (Folly et al., 2003), suggesting *T. rangeli* takes up host lipids *via* selective internalization of Lp. Lipid transport proteins (LTPs), another class of hemolymph proteins, are also a source of lipids in insects. LTPs serve a transfer function, mediating transport of lipids between fat bodies and Lp. The majority of lipid in the LTPs of the insects *M. sexta* (moth), *Periplanata americana* (cockroach), and *Locusta migratoria* (locust) are phospholipids and DAGs (Blacklock and Ryan, 1994). Similarly, another lipid carrier protein, called lipocalin, is also present in the digestive tract of the “kissing bug” *Rhodnius prolixus*, the vector of *Trypanosoma cruzi* (Ribeiro et al., 2014).

## 5 Fatty acid uptake in different organisms

**Figure 2:**
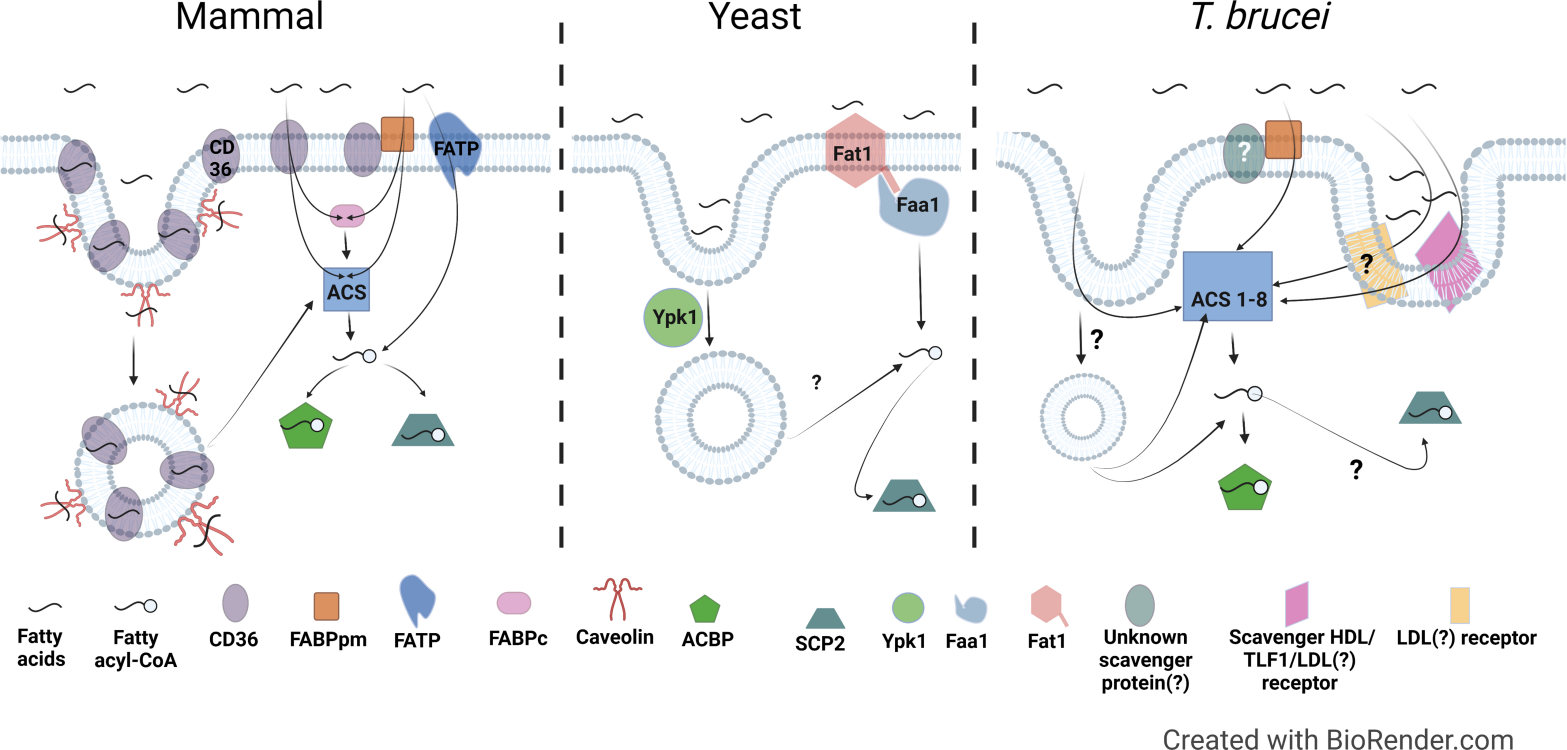
Comparison of fatty acid uptake machinery in mammals, yeast, and *T. brucei.* Left, Mammal: In mammals, the membrane proteins CD36, FABPpm, FATP are involved in fatty acid uptake either individually or in combination. CD36 and Caveolin are also involved in endocytosis mediated fatty acid uptake. CD36, Cluster of Differentiation 36; FABPpm, Plasma membrane associated fatty acid binding protein; FATP, Fatty acid transport protein; FABPc, Cytosolic fatty acid binding protein; ACS, Acyl-CoA Synthetase; ACBP, Acyl-CoA binding protein; SCP2, Sterol carrier protein 2. Middle, Yeast: Fat1, the ortholog of mammalian fatty acid transport proteins in yeast, is involved in fatty acid uptake. Faa1, which is a yeast ACS, activates the fatty acids by converting to fatty acyl-CoA. Ypk1, the yeast ortholog of the human serum- and glucocorticoid-induced kinase is involved in fatty acid uptake *via* endocytosis. Right, *T. brucei*: *T. brucei* possesses homologs to FABPpm, but presence or identity of FABPpm interacting partners is unknown yet. There is evidence of involvement of a scavenger receptor in uptake of HDL, LDL, and Trypanosome lytic factor 1 (TLF1) (Green et al., 2003), however, it is not clear if it is the same or different LDL receptor previously described by Coppens et al. (Coppens et al., 1992). Proteins involved in fatty acid uptake *via* endocytosis have not been identified yet. *T. brucei* genome encodes for 8 ACSs, which probably have different specificities to activate fatty acids of varying lengths and in different sub-cellular locations.

For cellular uptake of free NEFAs by passive diffusion, NEFAs must dissociate from carrier proteins such as albumin and either diffuse to the membrane prior to adsorption, or directly translocate into the membrane from the carrier protein-NEFA complex through membrane contact *via* random collision, ionic interactions, or possibly, facilitated by proteins (Vusse et al., 2022). Following adsorption to the membrane, the NEFAs then flip-flop to the inner leaflet and desorb from the membrane to intracellular acceptors. The rate limiting steps for NEFA uptake are transitioning the permeability barrier at the aqueous/lipid interface (adsorption/desorption) and flip-flop through the bilayer (Cupp et al., 2004; Simard et al., 2008; Carley and Kleinfeld, 2009; Arts et al., 2015; Vusse et al., 2022). Consequently, most eukaryotic cells rely on multiple mechanisms involving both membrane and soluble proteins to mitigate these rate limiting steps and facilitate diffusion to ensure a sufficient rate of fatty acid uptake (**Figure 2**) (Zhang et al., 2018; Wade et al., 2021). **Figure 2** summarizes the known (for mammals and yeast) and potential (for *T. brucei*) mediators of fatty acid uptake. **Table 1** summarizes known or putative proteins and pathways involved in fatty acid metabolism in mammals, yeast, and *T. brucei*.

**Table 1:** Fatty Acid Metabolism Proteins and Pathways.

Fatty acid metabolic pathway	Function(s)	Organism: protein involved	Protein name	Cellular location(s)
Fatty Acid Uptake	FA Partition into Membrane/Amino Acid Metabolism^a^	Mammals: FABPpm/mAspAT	Plasma Membrane Fatty Acid Binding Protein/Mitochondrial Aspartate Aminotransferase	Plasma Membrane, Mitochondrial Matrix
	Amino Acid Metabolism^a^	*T. brucei:* mASAT	Mitochondrial Aspartate Aminotransferase	Mitochondrial Matrix
	FA Partition into Membrane; FA Translocation across Membrane	Mammals: CD36	Cluster of Differentation 36	Plasma Membrane, Golgi, Endosomes
	FA Translocation across Membrane; FA Activation & Trapping	Mammals: FATP (n=6 in humans)	Fatty Acid Transport Protein	ER, Mitochondria, Peroxisomes, Plasma Membrane^b^
	FA Translocation across Membrane; FA Activation & Trapping	*S. cerevisiae*: FAT1	Fatty Acid Transport 1	Lipid droplets, Peroxisomes, Plasma Membrane
	FA Activation & Trapping	Mammals: ACS Family (n=26 in humans)	Acyl-CoA Synthetases	Endosomes, ER, Golgi, Lipid Droplets, Mitochondria, Peroxisomes, Plasma Membrane^b^
	FA Activation & Trapping	*T. brucei*: ACS Family (n=8)	Acyl-CoA Synthetases	None confirmed - See **Table 2**
	FA Activation & Trapping	*S. cerevisiae*: FAA (n=4)	Fatty Acid Activation (Acyl-CoA Synthetases)	Cytoplasm, Lipid Droplets, Peroxisomes, Plasma Membrane^b^
Soluble Fatty Acid Carriers	Intracellular Free FA Binding & Transport	Mammals: FABP (n=12 in humans)	Fatty Acid Binding Proteins	Cytoplasm, Nucleus^b^
	Intracellular Fatty Acyl-CoA Binding & Transport	Mammals: ACBP	Acyl-CoA Binding Protein	
	Intracellular Fatty Acyl-CoA Binding & Transport	*T. brucei:* ACBP (n=4)	Acyl-CoA Binding Protein	None confirmed - See **Table 2**
	Intracellular Free FA and Fatty Acyl-CoA Binding & Transport	*Y. lipolytica*: SCP-2	Sterol Carrier Protein 2	Peroxisome
	Not determined	*T. brucei*: SCP	Sterol Carrier Protein	None confirmed - See **Table 2**
**Fatty acid metabolic pathway**	**Function**	**Organism**	**Pathway name**	**Cellular location**
Fatty acid synthesis pathways	*De novo* Fatty Acid Synthesis	Mammals, Yeast	Type I Fatty Acid Synthase	Cytosol
	*De novo* Fatty Acid Synthesis	Mammals, Yeast, *T. brucei*	Type II Fatty Acid Synthase	Mitochondrial Matrix
	Fatty Acid Elongation	Mammals, Yeast	Microsomal Elongase	ER
	*De novo* Fatty Acid Synthesis	*T. brucei*	Microsomal Elongase	ER
Fatty acid oxidation pathways	Fatty Acid Degradation	Mammals	β-oxidation, α-oxidation, ω-oxidation	Peroxisomes (α,β), Mitochondria (β), ER (ω)
	Fatty Acid Degradation	Yeast	β-oxidation, α-oxidation, ω-oxidation	Peroxisomes (β,ω), ER(α)
	Fatty Acid Degradation	*T. brucei*	β-oxidation	Glycosomes

a FABPpm1 is a moonlighting function of mAspAT in mammals, where the same protein carries out both functions. In *T. brucei*, the FABPpm1 function and presence on the plasma membrane of Aspartate Aminotransferase (mASAT) has not been directly assessed.

b Multiple Locations depending on isoform.

### 5.1 Fatty acid uptake in mammals

#### 5.1.1 Cluster of differentiation 36

Cluster of Differentiation 36 (CD36) is an integral membrane protein in the scavenger receptor family with fatty acid translocase (FAT) activity that is involved in facilitated fatty acid uptake in various cell types (Abumrad et al., 1993; Glatz and Luiken, 2017; Glatz and Luiken, 2018) (**Figure 2**, left). In the plasma membrane, CD36/FAT accepts fatty acids into its extracellular domain, internalizing them through two putative entrances identified in its three dimensional structure (Hsieh et al., 2016). As the fatty acids diffuse into these entrances from the outer leaflet to the inner leaflet of the membrane bilayer, the fatty acids flip to orient themselves for translocation (Hamilton, 2007; Glatz and Luiken, 2018). However, the mechanism of how CD36/FAT facilitates fatty acid uptake is still not settled: an alternative view is that CD36 is not a translocase that facilitates diffusion, but instead mediates the partitioning of fatty acids into the membrane as part of passive diffusion (Pownall and Moore, 2014; Jay and Hamilton, 2018).

#### 5.1.2 Plasma membrane fatty acid binding protein

In certain mammalian cell types (e.g. hepatocytes, adipocytes, and cardiomyocytes), CD36 interacts with a peripheral membrane protein partner, the plasma membrane associated fatty acid binding protein (FABPpm) (**Figure 2**, left). FABPpm is secreted and associates with the extracellular face of the plasma membrane, where it is thought to assist fatty acid docking onto CD36 (Chabowski et al., 2007; Bradbury et al., 2011), although the exact mechanism of the CD36-FABPpm interaction is still being debated (Holloway et al., 2022). Both CD36 and FABPpm have been proposed as a potential facilitators for albumin-membrane interactions (Arts et al., 2015; Vusse et al., 2022). Interestingly, FABPpm appears to be a moonlighting protein, as it is apparently an isozyme of mitochondrial aspartate amino transferase (mASAT *aka* glutamic-oxaloacetic transaminase (GOT)) (Berk et al., 1990; Holloway et al., 2007; Bradbury et al., 2011) (**Table 1**). FABPpm was initially identified biochemically in rat liver and intestine as a ~40 kD membrane-associated fatty acid binding protein that facilitated trans-membrane transport of long-chain fatty acids (Stremmel et al., 1985; Stremmel et al., 1985). It was no doubt surprising to Berk et al. when an N-terminal peptide sequence of purified FABPpm matched the mitochondrial matrix protein mASAT (Berk et al., 1990). Yet, their extensive biochemical and immunological experiments led them to cautiously conclude that FABPpm and mASAT were “very similar” (Berk et al., 1990; Stump et al., 1993). Subsequent studies confirmed that mASAT and FABPpm are two functional forms of the same protein derived from the same mRNA (Bradbury and Berk, 2000). Subsequently, the site of fatty acid binding on FABPpm was mapped by molecular modeling and FABPpm function of mASAT confirmed by mutation (Bradbury et al., 2011). The mechanisms regulating the localization of FABPpm (i.e. plasma-membrane localized mASAT) and CD36 are not well-defined, but their translocation to the plasma membrane from intracellular pools (mitochondrial matrix (FABPpm) and ER or endosomes (CD36)) is known to be generally stimulated by insulin and by muscle contraction (Goldberg et al., 2009; Jain et al., 2009; Buqué et al., 2012).

#### 5.1.3 Fatty acid transport protein

Another group of proteins involved in mammalian fatty acid uptake are fatty acid transport proteins (FATP1–6 in humans) (**Table 1**). FATPs are integral membrane proteins that are members of the solute carrier superfamily SLC27A, which are present in various cellular locations including the plasma membrane, ER, mitochondria and peroxisomes (Kazantzis and Stahl, 2012; Zhan et al., 2012). Key to FATP function is their intrinsic acyl-CoA synthetase (ACS) activity, which catalyzes the ATP-dependent esterification of free fatty acids to CoA (Coe et al., 1999) (**Figure 2**, left). ACS activity is essential to the transport function of FATP (Stuhlsatz-Krouper et al., 1998; Zhan et al., 2012; Huang et al., 2021). However, it is not clear if FATP directly facilitates fatty acid diffusion into the cell, or if the ACS activity of FATPs can mediate net fatty acid uptake through metabolic trapping, whereby fatty acids enter the cell through passive diffusion and then are trapped inside by conversion to fatty acyl-CoAs. Metabolic trapping decreases the intracellular concentration of fatty acids, preventing efflux and maintaining the concentration gradient required for fatty acid transport (Martin et al., 1997). Complicating this simple model are findings that some FATPs harbor separable facilitated transport and fatty acid activation activities (Melton et al., 2011; Black et al., 2016). For example, a naturally-occurring splice variant of FATP2 that lacks ACS activity was found still to be competent for fatty acid transport (Melton et al., 2011; Black et al., 2016). A similar phenomenon was discovered in yeast FATP, whose ACS and transport functions could be mutationally separated (DiRusso et al., 2005). These observations suggest that some FATPs may have a distinct facilitative transport function independent of their ACS activity (Huang et al., 2021).

#### 5.1.4 Receptor-mediated endocytosis

Fatty acids are also taken up through receptor-mediated mechanisms (**Figure 2**, left). Receptor-mediated binding and cell-anchoring of lipoprotein particles can facilitate selective uptake of lipids, such as cholesteryl esters, directly into the cell without uptake of the whole particle (Seo et al., 2005). Conversely, uptake of whole serum lipoproteins by receptor-mediated endocytosis can provide fatty acids through breakdown of internalized phospholipid, TAGs, and cholesteryl ester cargo (Siddiqui et al., 2022). Albumin, along with its bound fatty acids, can also be internalized *via* a number of cell surface receptors, which direct albumin recycling, trafficking, and degradation (Sleep et al., 2013). Under normal conditions, Albumin is carrying only 0-1 fatty acids and so is unlikely to be a major contributor to overall fatty acid uptake. Caveolin 1 (Cav1), an important protein involved in caveolar endocytosis, also binds to fatty acids and mediates their internalization (Trigatti et al., 1999) (**Figure 2**, left). In addition, Cav1 in adipocytes is involved in fatty acid uptake *via* palmitoylation-regulated CD36-mediated caveolar endocytosis, delivering fatty acids to the ER, where the fatty acids are esterified then transported to lipid droplets for storage (Glatz and Luiken, 2018; Hao et al., 2020).’

#### 5.1.5 Acyl-CoA synthetases

Once fatty acids have entered the cell, they can be metabolically trapped by conversion to fatty acyl-CoAs either by FATPs or by soluble ACSs (**Figure 2**, left). Amongst a large family of more than 20 ACSs (Fernandez and Ellis, 2020), mammals have five long-chain ACS family members (ACSL1 and ACSL3–6), which collectively activate a range of fatty acids (C8–C22) (Quan et al., 2021) (**Table 1**). The ACSLs have distinct fatty acid specificities and sub-cellular localizations, including cytosolic and associated with the ER, endosomes, Golgi, lipid droplets, mitochondria, plasma membrane, and peroxisomes (Digel et al., 2009; Rossi Sebastiano and Konstantinidou, 2019). In addition, there are also ACSs specific for short chain, medium chain, and very long chain fatty acids. Fatty acid activation is necessary for fatty acids to undergo further catabolic or anabolic metabolism. Like FATPs, some ACSs can facilitate fatty acid uptake *via* metabolic trapping. For example, over-expression of ACSL5 stimulated C18:1 uptake and subsequent TAG synthesis in rat hepatoma cells (Mashek et al., 2006).

#### 5.1.6 Soluble binding proteins

Internalized non-esterified fatty acids also can bind to cytoplasmic Fatty Acid Binding Proteins (FABPs), a large family of soluble, predominantly cytoplasmic proteins distinct from FABPpm (Glatz and Luiken, 2018; McKillop et al., 2019). In addition, Acyl-CoA Binding Protein (ACBP) is a cytoplasmic protein that can bind fatty acyl-CoAs and traffic them to intracellular destinations such as ER, Golgi, and mitochondria (Knudsen et al., 2000; Hansen et al., 2008; Qiu and Zeng, 2020). Finally, Sterol Carrier Protein 2 (SCP-2), which is localized to both the cytoplasm and in peroxisomes (Keller et al., 1989; Gallegos et al., 2001), also can bind to a variety of lipids including fatty acids (Scallen et al., 1985; Stolowich et al., 2002). Collectively, FABPs, ACBP, and SCP-2 can retain and sequester intracellular fatty acids and acyl-CoAs to buffer the intracellular fatty acid load (Smathers et al., 2013) (**Figure 2**, left) (**Table 1**), and they can supply fatty acids and acyl-CoAs to downstream enzymes mediating various cellular processes like complex lipid synthesis, protein acylation, β-oxidation, and signaling (Schroeder et al., 1995; Færgeman and Knudsen, 1997; Knudsen et al., 2000).

### 5.2 Fatty acid uptake in yeasts

#### 5.2.1 Machinery in *Saccharomyces cerevisiae*

Yeasts have a less elaborate machinery for fatty acid uptake than mammals (**Figure 2**, middle) (Klug and Daum, 2014; Salvador López and Van Bogaert, 2021). Two FATP homologues, FAT1 and FAT2, have been identified in the budding yeast *Saccharomyces cerevisiae* (**Table 1**) (Færgeman et al., 1997; Jacquier and Schneiter, 2010; Salvador López and Van Bogaert, 2021). Fat1p is present in the plasma membrane, where it shows two distinct functions: import of fatty acids and their conversion into fatty acyl-CoA (Zou et al., 2002; DiRusso et al., 2005). Fat1p is also thought to be present in other cellular organelles like lipid bodies, the ER, and peroxisomes (van Roermund et al., 2012; Salvador López and Van Bogaert, 2021). Fat2p, also called Psc60p, is found in the peroxisomal lumen and is thought to be involved in the uptake of fatty acids from the cytosol into the peroxisome (Hiltunen et al., 2003).

*S. cerevisiae* also contains four ACSs named Fatty Acid Activation (FAA)1-4, each with a different fatty acid specificity (**Table 1**) (Knoll et al., 1994). Though Faa1 facilitates most cellular fatty acid uptake, Faa4 (but not Faa2p or Faa3p) could rescue the FAA1 deletion mutant, making Faa1p and Faa4p the most important ACSs, possibly with similar modes of action (Johnson et al., 1994). Faa1p, through its interaction with the carboxy terminus of Fat1p, has two distinct functions: transport and fatty acid activation to their respective CoA esters (**Figure 2**, middle) (Færgeman et al., 2001; Zou et al., 2002). Faa1p and Faa4p are localized to the plasma membrane and lipid droplets (Currie et al., 2014; Narita et al., 2016), and Faa2p is in the peroxisomes (Hettema et al., 1996). Faa3p may be at the cell membrane, but this has not been confirmed (Yeast GFP Fusion Localization Database - accessed on 10/6/2022; Huh et al., 2003).

Like mammals, yeasts also uptake fatty acids *via* endocytosis (**Figure 2**, middle). Deletion of YPK1, the yeast ortholog of the cytoplasmic human serum- and glucocorticoid-induced kinase (Sgk1), reduced fatty acid uptake in *S. cerevisiae* and exhibited a strong growth defect (Jacquier and Schneiter, 2010). Furthermore, the growth defect resulting from YPK1 deletion could be rescued by the addition of human Sgk1 kinase (Jacquier and Schneiter, 2010), indicating that the fatty acid transport function of this kinase is conserved between yeast and humans. Likewise, deletion of other proteins involved in endocytosis also impaired fatty acid uptake in yeast (Jacquier and Schneiter, 2010), suggesting endocytosis is an important pathway for fatty acid uptake in yeasts.

#### 5.2.2 Machinery in *Yarrowia lipolytica*

A single FAA1 and FAT1 homolog are also present in the oleaginous Ascomycete yeast *Y. lipolytica*, where they function in the utilization of exogenous fatty acids and *n-*alkanes (Tenagy et al., 2015). Faa1p is similarly involved in the activation of fatty acids to fatty acyl-CoAs, as deletion of FAA1 showed accumulation of saturated fatty acids in *Y. lipolytica*. In addition, Fat1p in *Y. lipolytica* has an additional function compared to its counterpart in *S. cerevisiae*, the transport of fatty acids from lipid droplets (Dulermo et al., 2014). Ascomycetes and Basidiomycetes also contain peroxisomal matrix SCP-2 homologs, which bind a variety of lipids including fatty acids and fatty acyl-CoAs (Tan et al., 1994; Edqvist and Blomqvist, 2006; Ferreyra et al., 2006). A number of functions have been proposed for these fungal SCP-2s, including facilitating peroxisomal β-oxidation (Niki et al., 1994; Edqvist and Blomqvist, 2006).

### 5.3 Fatty acid uptake in *T. brucei*

#### 5.3.1 Strategies to mobilize environmental lipids

As detailed above, in the mammalian blood, lymph, and interstitial fluids, most NEFAs are bound by serum albumin (Lee and Wu, 2015; De Simone et al., 2021). There are three possible ways *T. brucei* mobilizes albumin-bound fatty acids. One way is by fluid-phase endocytosis of albumin (Coppens et al., 1987; Engstler et al., 2004). However, the 200-fold lower clearance rate of albumin compared to transferrin, which is taken up *via* receptor-mediated endocytosis, suggests this route is a minor contributor to overall fatty acid uptake. A second way is that albumin may dock with the parasite surface and then fatty acids are abstracted directly into the plasma membrane (Elmadhoun et al., 2012; Vusse et al., 2022). There is conflicting evidence for stable albumin binding to the BSF surface (Diffley, 1978; Coppens et al., 1987; Webster, 1989; Vandeweerd and Black, 1990). When detected, bound albumin was only 0.2% of total soluble proteins, indicating it may be a minor contributor to fatty acid uptake. However, transient docking interactions between albumin and the parasite surface have not been ruled out. A third way, likely the main route of fatty acid mobilization from albumin, is that fatty acids dissociate from albumin prior to their adsorption to the parasite plasma membrane (Voorheis, 1980; Demant et al., 2002; Vusse et al., 2022). This idea is supported by the fact that *T. brucei* can readily take up and metabolize NEFAs provided in the absence of albumin (Doering et al., 1993).

*T. brucei* BSFs secrete a highly active lysophospholipase A (LysoPLA), which releases the single fatty acid from extracellular *lyso*-phospholipids, either bound to albumin or present in serum lipoproteins (Samad et al., 1988; Vandeweerd and Black, 1990; Bowes et al., 1993). This lysoPLA appears to work in concert with a membrane-associated acyl-CoA ligase and acyltransferase to take the fatty acid liberated from lyso-phospholipids, mainly lyso-phosphatidylcholine (LPC) or *lyso*-phosphatidylethanolamine (LPE), activate it to an acyl-CoA, and then transfer the acyl group to a second LPC or LPE to generate a fully-acylated phosphatidylcholine and phosphatidylethanolamine within the plasma membrane (Mellors and Samad, 1989; Bowes et al., 1993).This pathway provides an efficient route to mobilize exogenous *lyso*-phospholipids, and their fatty acids such as C14:0. For example, [^3^H]C14:0 (myristic acid) was shown to be hydrolyzed extracellularly from[^3^H]myristoyl-LPC, taken up, and incorporated into the VSG GPI-anchor nearly as efficiently as free [^3^H]myristic acid (Werbovetz and Englund, 1996). For further reading on phospholipid, sphingolipid, and neutral lipid metabolism in *T. brucei*, please consult these reviews (Smith and Bütikofer, 2010; Serricchio and Bütikofer, 2011; Parreira de Aquino et al., 2021).

#### 5.3.2 Previous biochemical characterization of fatty acid uptake in *T. brucei*

Early studies showed that *T. brucei* can readily take up saturated and unsaturated long chain fatty acids from its environment (Dixon et al., 1971; Voorheis, 1980). These studies looked at uptake of radiolabeled C16 and C18 fatty acids pre-equilibrated with albumin at final concentrations of 20-40 µM. Uptake was biphasic, with an initial rapid phase (<5 sec.), likely reflecting the initial adsorption of fatty acids to the membrane, followed by a slower phase of uptake reflecting fatty acid internalization and subsequent metabolism (Voorheis, 1980). Both studies indicated that PCFs had greater extent of fatty acid uptake than BSFs (up to 15-fold higher). The kinetics and extent of uptake also varied depending on the fatty acid. In general, uptake of C16:0, C18:2 and C18:3 was greater than C18:0 and C18:1. PCFs in particular, exhibited a very robust uptake of C18:3 (linolenic acid) (Dixon et al., 1971). Uptake of some fatty acids (C16:0, C18:2, and C18:3) appeared saturable in BSFs, raising the possibility that protein-mediated processes were involved (Dixon et al., 1971). Moreover, fatty uptake was reversible, in that 75% of the radiolabeled fatty acids internalized during a 15 min. incubation could be back-extracted by a 15 min. chase with apo-albumin, indicating that internalized fatty acids weren’t immediately metabolized and remained exchangeable with the environment.

The discovery that the *T. brucei* BSF GPI-anchor was exclusively myristoylated (Ferguson and Cross, 1984; Ferguson et al., 1985) triggered an interest in the uptake and metabolism of C14:0 and other medium chain fatty acids by *T. brucei*. BSF *T. brucei* could take up C14:0 from the environment and use it for GPI biosynthesis (Doering et al., 1993; Werbovetz and Englund, 1996). PCFs also took up C14:0 and used it for GPI biosynthesis, but the C14:0 was elongated to C16:0 and C18:0 prior to its incorporation into the procyclin GPI-anchors (Field et al., 1991; Bütikofer et al., 1997). In BSFs, uptake of [^3^H]C14:0 (in presence of 5% calf serum) was linear over 60 min. for concentrations 1–12 µM (Doering et al., 1993), but subsequent incorporation of C14:0 into proteins and lipids started to plateau over the same time frame at concentrations of 2-9 µM, suggesting that the pathways utilizing C14:0 are saturable (Krakow et al., 1986). Despite their need for C14:0, BSFs had higher uptake of C16 and C18 long-chain fatty acids compared to medium chain fatty acids (C10–C14) (Mellors and Samad, 1989; Morita et al., 2000). Moreover, as the fatty acid chain-lengths got shorter, the extent of uptake trended lower until negligible uptake was observed with fatty acids <C8:0 (Morita et al., 2000). The extent to which these features and preferences are encoded by the fatty acid uptake machinery or in the downstream fatty acid metabolic pathways is unknown. However, C16:0 and C18:0 both competed with C14:0 for uptake in BSFs when co-incubated at their relative physiological total serum lipid concentrations (25 µM and 2 µM, respectively) (Doering et al., 1993). Because the subsequent metabolic fate of C14:0 is quite different from the fate of C16:0 and C18:0 (GPI-biosynthesis vs. phospholipid synthesis, respectively), this competition suggests that C14:0 is likely taken up by the same machinery as the long chain fatty acids C16:0 and C18:0.

#### 5.3.3 Candidate fatty acid machinery in *T. brucei*

How might *T. brucei* facilitate uptake of fatty acids across its plasma membrane? Using a Basic Local Alignment Search Tool (BLAST) search of the *T. b. brucei* TREU927 genome (Berriman et al., 2005) in TriTrypDB (Amos et al., 2022) for homologs to known fatty acid uptake factors revealed that *T. brucei* appears to have a distinct repertoire of potential fatty uptake machinery (**Figure 2**, right, and **Table 2**). *T. brucei* lacks predicted homologs to mammalian and yeast FATPs and to mammalian caveolin and CD36/FAT, main plasma membrane transporters for fatty acid uptake in other organisms.

**Table 2:** Putative *T. brucei* Homologs to Known Fatty Acid Uptake Proteins.

Fatty acid uptake proteins	*T. b. brucei* Homolog(s)	GeneID (TREU 927) (https://tritrypdb.org/tritrypdb/app)^c^	Prior published work on these genes/proteins in *T. brucei*	TrypTag localization (http://tryptag.org)^d^NT: N-term tag CT: C-term tag
Acyl-CoA Synthetases	Acyl-CoA Synthetase 1 (ACS1)	Tb927.9.4190	(Jiang et al., 2000; Jiang & Englund, 2001; Jiang et al., 2004)	NT & CT: cytoplasm
	Acyl-CoA Synthetase 2 (ACS2)	Tb927.9.4200		NT: cytoplasm CT: endoplasmic reticulum (ER)
	Acyl-CoA Synthetase 3 (ACS3)	Tb927.9.4210		NT: cytoplasm, nucleoplasm
	Acyl-CoA Synthetase 4 (ACS4)	Tb927.9.4230		NT: cytoplasm
	Long-Chain Acyl-CoA Synthetase 5 (LACS5), putative	Tb927.10.3260		NT: cytoplasm, nucleoplasm CT: flagellar pocket membrane, pellicular membrane, ER
	Fatty Acyl-CoA Synthetase, putative (ACS6^a^)	Tb11.v5.0561		Not tagged/Low priority gene (no proteomic evidence for expression)
	Fatty Acyl-CoA Synthetase, putative (ACS7^a^)	Tb11.v5.0825		Not tagged/Low priority gene (no proteomic evidence for expression)
	Long-Chain Fatty Acid CoA Ligase, putative (ACS8^a^)	Tb927.11.7530		NT: cytoplasm CT: mitochondrion, kinetoplast
Plasma Membrane Fatty Acid Binding Protein (FABPpm1)^b^	Mitochondrial Aspartate Amino-transferase (mASAT)^b^	Tb927.11.5090	(Marciano et al., 2008; Abendroth et al., 2015)	NT: cytoplasm CT: cytoplasm, flagella, kinetoplast Biochemical evidence for mitochondrial localization (Marciano et al., 2008)
Acyl-CoA Binding Protein (ACBP)	Acyl-CoA Binding Protein, putative	Tb927.4.2010	(Milne & Ferguson, 2000; Milne et al., 2001)	NT: glycosome
	Acyl-CoA Binding Protein, putative	Tb927.7.6770		NT & CT: cytoplasm, flagella, nucleoplasm
	Acyl-CoA Binding Protein, putative	Tb927.11.12830		NT & CT: cytoplasm
	Acyl-CoA Binding Protein, putative	Tb927.11.352		NT: Golgi apparatus
Sterol Carrier Protein 2 (SCP2)	Sterol Carrier Protein, putative	Tb927.8.1820		NT: cytoplasm CT: mitochondrion, kinetoplast
Cellular Fatty Acid Binding Proteins (cFABP)	*None*			
CD36/FAT	*None*			
Caveolin	*None*			

a Proposed name.

b FABPpm1 is a moonlighting function of mASAT in mammals. This moonlighting function has not been investigated in T. brucei.

c (Amos et al., 2022).

d (Dean et al., 2017; Billington et al., 2022).

##### 5.3.3.1 FABPpm

The only candidate plasma membrane-associated fatty acid uptake protein present in the *T. brucei* genome is the mitochondrial aspartate amino transferase (mASAT), whose moonlighting function as FABPpm is well-documented in mammals [recently reviewed in (Mallick et al., 2021)]. The mitochondrial aspartate aminotransferase function of this enzyme has been previously characterized in *T. brucei* (Marciano et al., 2008; Abendroth et al., 2015), but the FABPpm moonlighting function has not been directly investigated. To date, there is no evidence for mASAT being localized to the plasma membrane in *T. brucei*: C-terminal fluorescent-tagging studies indicate mitochondrial (kinetoplast) and cytosolic labeling of tagged mASAT (Tryptag.org: Dean et al., 2017, Billington et al., 2022), and no mASAT peptides have been detected in surface proteomic studies (Oberholzer 2011, Gadelha 2015, Shimogawa 2015). However, the plasma membrane pool of mASAT may be difficult to detect compared to the total cellular pool without enrichment. Thus, the extent to which *T. brucei* mASAT functions as a FABPpm to facilitate fatty acid uptake is unknown. In mammals, the most important partner of FABPpm in fatty acid uptake is thought to be CD36, as it has been shown that CD36 has an additive effect on fatty acid uptake if co-expressed with FABPpm (Holloway et al., 2022). As CD36-encoding genes have not been identified in *T. brucei*, if *T. brucei* mASAT does function as FABPpm, it is also not clear if there is another scavenger receptor partner to FABPpm that awaits identification.

##### 5.3.3.2 ATP-Binding Cassette (ABC) transporters

Another class of integral membrane transporters are the ATP-Binding Cassette (ABC) transporters, a large family of dimeric transmembrane proteins that couple transport with ATP hydrolysis (Tarling et al., 2013; Wilkens, 2015). A subset of ABC transporters that can transport fatty acids and/or fatty acyl-CoAs have been reported in yeast, plants, and humans (Verleur et al., 1997; van Roermund et al., 2008; Kim et al., 2013; Cai et al., 2021), where they mediate fatty acid import into peroxisomes for their degradation by β-oxidation. ABC transporters also have been identified in *T. brucei* (Mäser and Kaminsky, 1997), with ~20 ABC transporters annotated in the genome (Amos et al., 2022). Among them are three glycosomal ABC transporters (GAT1–3) (Yernaux et al., 2006; Igoillo-Esteve et al., 2011). GAT1 can transport C18:1-CoA, and its downregulation by RNA interference (RNAi) caused a significant reduction of [^14^C]C18:1-CoA uptake by glycosomes in PCFs with a concomitant accumulation of cytosolic C18:2 (the product of further 18:1 desaturation) and other unsaturated fatty acids (Igoillo-Esteve et al., 2011). The substrate preferences of GAT1 and its effect on total cellular fatty acid uptake haven’t been fully defined, but it is possible that the glycosomal GAT1 (and their peroximal counterparts) may facilitate cellular fatty acid import indirectly by reducing the cytosolic fatty acid/acyl-CoA concentration.

##### 5.3.3.3 SCP-2 and ACBP

In terms of intracellular proteins potentially mediating fatty acid uptake, there are no predicted homologs to cytoplasmic FABPs, but *T. brucei* has homologs to two other members of the intracellular lipid binding protein family, SCP-2 and ACBP. *T. brucei* SCP-2 has not been functionally characterized and its cellular localization is unclear (**Table 2**), but the related protein SCP2-thiolase (SLP; Tb927.8.2540) functions in sterol synthesis in PCFs (Harijan et al., 2016). *T. brucei* possesses four ACBP homologs. One of the ACBPs (Tb927.4.2010) was shown to be essential in *T. brucei* BSFs, where it preferentially bound medium chain acyl-CoAs and stimulated VSG GPI-anchor synthesis in a cell-free assay (Milne and Ferguson, 2000; Milne et al., 2001). The other ACBP homologs have not been studied, and their predicted cellular localizations (**Table 2**) remain to be confirmed. Like Tb927.4.2010 ACBP, these other candidate ACBPs may potentially play similar roles in acyl-CoA transport and subsequent metabolism.

##### 5.3.3.4 ACSs

Finally, *T. brucei* has eight predicted ACSs. Four ACSs (ACS1–4) are in a tandem array on Chromosome 9 (Jiang et al., 2000). The enzymatic activities of purified recombinant ACS1–4 were characterized (Jiang and Englund, 2001). ACS1, -3 and -4 had distinct but overlapping specificities for fatty acyl chain-length and unsaturation, while ACS2 activity was more restricted to medium chain C10:0-CoA and C12:0-CoA substrates. Interestingly, ACS1, -3, and -4 all were able to use C14:0 as a substrate, indicating metabolic redundancy for activation and intracellular trapping of C14:0. ACS3 (and possibly some ACS4, which is 95% identical to ACS3) was the main ACS activity purified from BSF membranes (Jiang et al., 2004). The remaining four predicted ACSs have not been functionally characterized. Moreover, the sub-cellular localizations of all eight ACSs (**Table 2**) remain to be confirmed, but the variety of reported localizations in the Tryptag database (Dean et al., 2017; Billington et al., 2022) suggest that ACSs may have distinct functions in different parts of the cell.

##### 5.3.3.5 Receptor-mediated endocytosis in BSFs

BSFs express high levels of clathrin (Morgan et al., 2001) and thus exhibit a very high rate of clathrin-mediated endocytosis, recycling the entire VSG coat every 12 min. (Allen et al., 2003; Engstler et al., 2004; Overath and Engstler, 2004). As mentioned above, *T. brucei* BSFs can take in limited amounts of albumin-bound lipids through fluid-phase endocytosis (Coppens et al., 1987; Overath and Engstler, 2004). *T. brucei* also acquires fatty acids from serum lipoprotein-associated lipids (phospholipids, sphingolipids, and cholesteryl esters) through receptor-mediated endocytosis of LDLs and HDLs (Vandeweerd and Black, 1990; Coppens et al., 1995; Green et al., 2003). Multiple candidates for the *T. brucei* receptors for the mammalian lipoproteins have been purified and/or biochemically characterized in BSFs (Coppens et al., 1991; Gillett and Owen, 1992; Green et al., 2003). Sowing confusion about these receptors is that they potentially have over-lapping affinities for LDL and HDL, and their genetic identities remain unknown. These receptors are distinct from the haptoglobin-hemoglobin receptor, which binds the trypanolytic HDL species Trypanolytic Factor 1 and -2 (Vanhollebeke et al., 2008; Capewell et al., 2015). Having multiple broadly-specific receptors for host apolipoproteins could be adaptive for the parasite in terms of efficiently acquiring the abundant host lipids present in their lipoproteins. After internalization, fatty acids are liberated upon Apolipoprotein B-100 degradation in the endo-lysosome, which destabilizes the lipoprotein particle (Coppens et al., 1995; Coppens and Courtoy, 2000), although some release of lipids also may occur in the flagellar pocket (Vandeweerd and Black, 1990).

##### 5.3.3.6 Receptor-mediated endocytosis in PCFs

PCFs also undergo fluid-phase and receptor-mediated endocytosis, but at a much lower rate than in BSFs (Langreth and Balber, 1975; Webster, 1989). *T. brucei*-Lp interactions have not been investigated. However, using Lp purified from the kissing bug *R. prolixus*, studies showed that two different trypanosomatids (*T. rangeli* and *Herpetomonas muscarum*) were able to bind to and take up Lp, likely by receptor-mediated endocytosis (Folly et al., 2003; Kluck et al., 2018). Moreover, both trypanosomatids could acquire Lp-associated lipids, either [^3^H]C16:0 (Kluck et al., 2018) or fluorescently-tagged phospholipids (Folly et al., 2003). Finally, the *T. brucei* PCF-specific Cysteine-Rich Acidic trans-Membrane protein (CRAM) acts as receptor for HDL in PCFs, though other HDL uptake pathways were detected (Liu et al., 2000). This HDL uptake pathway may be physiologically relevant, because PCFs likely encounter HDL in the bloodmeal. Alternatively, HDL may not be the native substrate for CRAM and it’s possible CRAM may mediate uptake of Lp or other insect lipoproteins. Finally, *T. brucei* does not possess a predicted homolog of caveolin, which is restricted to metazoans (Kirkham et al., 2008), and thus does not acquire fatty acids through caveolin-mediated mechanisms.

#### 5.3.4 A speculative model of fatty acid uptake in *T. brucei*

Based on the available published observations and the predicted homologs present in the genome, we propose a speculative model for fatty acid uptake in *T. brucei* (**Figure 2**, right). The potential fatty acid uptake machinery in *T. brucei* is distinct from both mammals and yeast (**Tables 1**, **2**). *T. brucei* appears to lack trans-membrane transport proteins like FATP, instead possessing only an FABPpm, without an obvious CD36-like partner. FABPpm may be able to assist fatty acids in partitioning into the membrane and/or abstracting fatty acids from albumin. Given the lack of CD36/FAT and FATP homologs, it is possible, even likely, that the bulk of fatty acid transit across the plasma membrane is unfacilitated in *T. brucei*, occurring instead by passive diffusion. Once fatty acids are internalized, *T. brucei* possesses eight ACSs to activate and trap the fatty acids inside the cells, far fewer than in mammals but more than in yeasts. These fatty acyl-CoAs could be metabolized further or be bound by ACBPs and/or SCP-2, the latter of which may also be able to bind free fatty acids. ACBP and SCP-2 may sequester the fatty acids/acyl-CoAs and/or facilitate their delivery to subsequent metabolic pathways. Finally, in addition to the diffusion and trapping pathway described above, endocytic uptake of host lipoproteins and to a lesser extent, mammalian albumin, likely serves as a second major pathway for *T. brucei* to acquire host fatty acids.

## 6 Final thoughts

### 6.1 Fatty acid uptake and synthesis are two sides of the same coin

Myristic acid is concentrated almost exclusively in the BSF GPI-anchor pool and in BSFs freshly isolated from blood, ~88% of cellular C14:0 was associated with VSG (Doering et al., 1993). In *T. brucei* BSFs, the fate of internalized C14:0 depended upon the level of lipids in the environment. When *T. brucei* was incubated with [^3^H]C14:0 in lipid-poor saline buffer, most of the labeled C14:0 was elongated and incorporated into phospholipids. However, when [^3^H]C14:0 labeling was performed in the presence of unlabeled C16:0 or in whole blood, C14:0 was not elongated and instead was incorporated into the VSG GPI-anchors (Doering et al., 1993). Similarly, when *T. brucei* was labeled with [^3^H]C14:0 in whole blood, there was no elongation of the radiolabeled fatty acid and it was directly incorporated into GPI-anchors, suggesting that under an adequate supply of longer fatty acids, C14:0 is preferentially channeled to the GPI-anchor biosynthesis pathway, rather than entering either the fatty acid elongation or phospholipid synthesis pathways (Doering et al., 1993). Given that most tissues of the mammalian host provide an abundant supply of long chain fatty acids, either in an unesterified form or as part of more complex lipids, BSF *T. brucei* has specialized its fatty acid synthesis machinery to produce C14:0 that is primarily used for VSG GPI-anchor synthesis. This specialization of fatty acid synthesis for C14:0 for the VSG GPI-anchors only works if the parasite can satisfy its other fatty acid needs through uptake from the host. Therefore, fatty acid uptake and synthesis are two sides of the same coin and trypanosomes likely need both to survive.

### 6.2 Fatty acids as building blocks or energy sources?

Fatty acids that are taken up can be used as building blocks for biosynthesis of more complex lipids. Alternatively, fatty acids can be catabolized by various fatty acid oxidation pathways, which typically will drive ATP production *via* oxidative phosphorylation. *T. brucei* appears to be capable of carrying out β-oxidation reactions, but it may occur only in specific environments, such as the adipose tissue (Dixon et al., 1971; Wiemer et al., 1996; Allmann et al., 2014; Trindade et al., 2016). Fatty acid β-oxidation as a source for energy in *T. brucei* has not been experimentally confirmed to date. It is important to note that β-oxidation of fatty acids also can be used for chain shortening (i.e. fatty acid remodeling) as a part of lipid biosynthesis as well as contributing to energy production. Below, we consider the possible contribution of host-acquired fatty acids as a carbon source in different host tissues.

In mammals, blood glucose is highly regulated, with a concentration in the range of 4.4-11.1 mM (Maggs et al., 1995; Thomas et al., 1998; Egi et al., 2006; Egi et al., 2008; Lozano et al., 2020), and thus represents an abundant carbon source in that tissue that BSFs readily use for energy generation *via* glycolysis and substrate-level phosphorylation [recently reviewed in (Smith et al., 2017)]. Although BSFs readily take up fatty acids, they do not appear to oxidize them (Dixon et al., 1971; Doering et al., 1993; Trindade et al., 2016), so we speculate that BSFs likely take and use host fatty acids as biosynthetic building blocks for more complex lipids and protein anchors. For the skin, the glucose concentration in the subcutaneous tissues of the arm and abdomen were on the order of 88-97% and 84-87%, respectively, of serum glucose levels (Thomas et al., 1998). This abundance of glucose suggests it is likely the preferred carbon source for *T. brucei* in skin also. In CSF, the reported glucose concentrations are highly variable, ranging from 44-90% in normal subjects, depending upon age and other conditions (Nigrovic et al., 2012; Hegen et al., 2018; Shahan et al., 2021). Although glucose is lower in CSF, it is relatively abundant compared to fatty acid concentrations, which are even more depleted in CSF [on the order of 0.5% of plasma levels (Koch et al., 2001)]. So we predict that fatty acids are unlikely to be a good energy source in the CSF. However, the energy metabolism and fatty acid uptake capacity of *T. brucei* dwelling in skin and CSF have not been investigated, so the carbon source preferences of *T. brucei* in these tissues remain unclear.

In contrast to other mammalian tissues, the glucose concentration in adipose is much lower, <15% compared to that in the plasma (Maggs et al., 1995). Studies on the metabolism of BSFs in adipose showed they have upregulated RNA expression of fatty acid β-oxidation genes and they have β-oxidation enzymatic activity (Trindade et al., 2016). However, in the mammalian host, *T. brucei* lacks a functioning TCA cycle and oxidative phosphorylation pathway, and so energy generation from β-oxidation could not follow the traditional pathway of Acetyl-CoA oxidation by the TCA cycle and ATP generation by the F_o_F_1_ ATP Synthase. Instead, it was recently proposed that adipose BSFs might use β-oxidation to shorten and remodel host-acquired fatty acids prior to their incorporation into more complex lipids, while the released Acetyl-CoA contributes to ATP production *via* substrate-level phosphorylation through the acetate:succinate CoA transferase (Smith et al., 2017). Along with fatty acids, glycerol is also released by adipose tissue during lipolysis. Reported adipose interstitial glycerol concentrations are in the range of 240-2800 µM, which is 3 to 31-fold higher than serum glycerol concentrations (Maggs et al., 1995; Li et al., 2006; Vestergaard et al., 2013). Glycerol is also present in the dermis, ~40-50 µM (Boschmann et al., 2001). Although BSFs prefer glucose, they can oxidize glycerol for ATP production and even adapt to survive on glycerol as the sole carbon source in the absence of glucose (Pineda et al., 2018). Glycerol use has not been assessed in adipose or skin-dwelling *T. brucei*, but the abundance of glycerol and the evidence for mixed use of glucose and glycerol suggests that glycerol, along with glucose, may serve as a potential carbon source for energy production in these tissues.

In the tsetse fly, *T. brucei* switches its energy metabolism from glucose oxidation to amino acid oxidation, primarily of proline, which is abundant in tsetse fly hemolymph at 1-2 mM (Balogun, 1974; Bringaud et al., 2006). This switch in carbon source preference is established by the time the parasites reach the proventriculus, and is required for survival in the tsetse host (Mantilla et al., 2017; Naguleswaran et al., 2021). Prior to that shift, PCFs retain a high degree of metabolic flexibility, able to use a variety of carbon sources (in order of preference): glycerol, glucose, and proline (Coustou et al., 2008; Allmann et al., 2021; Naguleswaran et al., 2021). In most insects, trehalose is the dominant hemolymph sugar and is more abundant than glucose (28 mM vs. 58 mM in one study in the fruit fly *Drosophila melanogaster* (Song et al., 2010). Trehalose has been detected in tsetse flies (Geigy et al., 1959) but neither glucose nor glycerol levels have been measured in tsetses, though glucose is widely presumed to be low. In other insects such as centipedes, earwigs, and dragonflies, very little glucose was found: the highest concentration among these was in dragonfly, which was less than 0.5 mM (Bedford, 1977). The higher hemolymph glucose concentration reported in Drosophila may be due to its 10% sucrose diet, so may not reflect normal physiological levels in obligate hematophagous insects like tsetse flies. Like BSFs, PCFs readily take up fatty acids, but available biochemical and genetic evidence suggest that PCFs do not appear to carry out significant β-oxidation (Dixon et al., 1971; Allmann et al., 2014). Thus, we speculate that in the midgut, which is periodically replenished with a blood meal, the PCFs rely on metabolism of glycerol, glucose, and proline for energy, and use acquired fatty acids for biosynthetic purposes. The energy metabolism and fatty acid uptake in the other developmental stages in the tsetse (proventricular forms, epimastigotes, and metacyclics) are not well understood, but proline oxidation is clearly important. However, it is possible that fatty acid oxidation may contribute to energy production in these other developmental stages, but confirmation of this awaits future studies.

### 6.3 Exploiting fatty acid uptake for therapeutic purposes

Understanding the role of fatty acid uptake and synthesis in *T. brucei* not only helps to understand overall fatty acid metabolism in this parasite, but this knowledge also can be beneficial for development of new HAT and AAT treatments. For example, a myristate analog, 11-oxatetradecanoic acid (O-11), was selectively toxic to BSF but not PCF *T. brucei*, likely because it disrupted VSG GPI-anchor biosynthesis, which is a major consumer of myristate for GPI-remodeling (Doering et al., 1991; Doering et al., 1994). Though promising in culture and showing no apparent cytotoxicity towards the host, the O-11 myristate analog was ineffective in a mouse model of infection, apparently due to poor pharmacodynamics (Werbovetz et al., 1996). Likewise, targeting one or both of the fatty acid uptake and *de novo* fatty acid synthesis machineries also could constitute a potential novel therapeutic target against *T. brucei* and other African trypanosomes (Stephens et al., 2007; Steketee et al., 2021). Finally, the parasitic fatty acid uptake machinery could also be potentially exploited for new lipid-based drug delivery systems in the treatment of HAT and AAT (Shrestha et al., 2014; Fattahi et al., 2020). Compounds, in the form of pro-drugs, could be conjugated to fatty acids and loaded into liposomes or lipid nanoparticles for increased stability, and their compositions potentially “tuned” for optimal uptake by *T. brucei*. The *in vivo* efficacy of O-11 myristate analogs may be significantly enhanced through just such a strategy, an idea worth revisiting. Finally, these lipophilic compounds can be adapted for trans-dermal drug delivery, which raises the exciting possibility of treating *T. brucei* infections harboring in the skin (Chacko et al., 2020).

## Author contributions

NP and KP conceived and wrote the manuscript. NP prepared the figures. NP and KP compiled the Table. Both authors contributed to the article and approved the submitted version.

## Funding

This work was supported by funds from the National Institute of General Medicine (NIGMS) to KP as a COBRE Target Investigator (P20GM109094; PI: L.Temesvari).

## Conflict of interest

The authors declare that the research was conducted in the absence of any commercial or financial relationships that could be construed as a potential conflict of interest.

## Publisher’s note

All claims expressed in this article are solely those of the authors and do not necessarily represent those of their affiliated organizations, or those of the publisher, the editors and the reviewers. Any product that may be evaluated in this article, or claim that may be made by its manufacturer, is not guaranteed or endorsed by the publisher.
